# The Medical News Pocket Formulary, New (4th) Edition

**Published:** 1902-02

**Authors:** 


### Books and Magazines.

---

The Medical News Pocket Formulary, New (4th) Edition.—Containing 1700 prescriptions representing the latest and most approved methods of administering remedial agents. By E. Quin Thornton, M. D., Demonstrator of Therapeutics, Pharmacy and Materia Medica in the Jefferson Medical College, Philadelphia. New (4th) edition, carefully revised to date of issue. In one wallet-shaped volume, strongly bound in leather, with pocket and pencil. Price, $1.50, net. Lea Brothers & Co., Philadelphia and New York. 1902.

The appearance of four editions in as many years testifies to the popularity of Dr. Thornton's formulary.

The present edition contains all of the new remedies and a number of new contributions of the older drugs. The method of using hydrogen peroxide to remove powder stains and of using adrenalin as a hæmostatic, together with a large number of new formulæ, have been added.

The diseases are alphabetically arranged and the indications for the use of the various formulæ are appended. R.

